# Congruent aero-tactile stimuli bias perception of voicing continua

**DOI:** 10.3389/fnhum.2022.879981

**Published:** 2022-07-15

**Authors:** Dolly Goldenberg, Mark K. Tiede, Ryan T. Bennett, D. H. Whalen

**Affiliations:** ^1^Haskins Laboratories, New Haven, CT, United States; ^2^Department of Linguistics, University of California, Santa Cruz, Santa Cruz, CA, United States; ^3^The Graduate Center, City University of New York (CUNY), New York, NY, United States; ^4^Department of Linguistics, Yale University, New Haven, CT, United States

**Keywords:** sensory integration, action-perception, multimodal speech perception, perceptual units, tactile perception

## Abstract

Multimodal integration is the formation of a coherent percept from different sensory inputs such as vision, audition, and somatosensation. Most research on multimodal integration in speech perception has focused on audio-visual integration. In recent years, audio-tactile integration has also been investigated, and it has been established that puffs of air applied to the skin and timed with listening tasks shift the perception of voicing by naive listeners. The current study has replicated and extended these findings by testing the effect of air puffs on gradations of voice onset time along a continuum rather than the voiced and voiceless endpoints of the original work. Three continua were tested: bilabial (“pa/ba”), velar (“ka/ga”), and a vowel continuum (“head/hid”) used as a control. The presence of air puffs was found to significantly increase the likelihood of choosing voiceless responses for the two VOT continua but had no effect on choices for the vowel continuum. Analysis of response times revealed that the presence of air puffs lengthened responses for intermediate (ambiguous) stimuli and shortened them for endpoint (non-ambiguous) stimuli. The slowest response times were observed for the intermediate steps for all three continua, but for the bilabial continuum this effect interacted with the presence of air puffs: responses were slower in the presence of air puffs, and faster in their absence. This suggests that during integration auditory and aero-tactile inputs are weighted differently by the perceptual system, with the latter exerting greater influence in those cases where the auditory cues for voicing are ambiguous.

## Introduction

In multisensory (or multimodal) integration, information from different sensory modalities, such as sight, audition, or somatosensation, are integrated by the human perceptual and nervous system into a coherent percept (see Rosenblum, 2008a; Stein and Stanford, 2008; Stein et al., 2009; Spence and Bayne, 2015; for review and discussion). This integration occurs even though the input from different sensory modalities is processed at different speeds (Eagleman, 2008): for instance, auditory input reaches the cortex in less than half the time of visual input (Molholm et al., 2002). Animal studies with single neurons indicate that there are differences in the way that multimodal and unimodal signals are processed (Stein and Stanford, 2008), consistent with human use of separate, multimodal regions for some tasks (e.g., Banati et al., 2000; Calvert, 2001). Direct comparisons of neural processing speeds for haptic input are more difficult, since possible contact points on the skin are distributed over the entire body, not just the area of the eyes and ears. To complicate matters further, the speed of processing is affected by factors such as stimulus intensity (e.g., Colonius and Diederich, 2004), previous experience (Miyazaki et al., 2006), or the way stimuli are presented (Harrar and Harris, 2008), all of which can affect the salience of correspondence between different sensory inputs during the process of integration.

A relevant question is how sensations associated with different afferent timings become integrated and perceived as a single coherent event. One possibility could be a dynamic recalibration of expectations. Eagleman and Holcombe (2002) and Haggard et al. (2002) demonstrated that participants perceive two events from different modalities (haptic and visual, in this case) as being closer temporally than they are in fact because they perceive them as part of the same event: a flash of light that appeared after the participants have pressed a button was perceived as immediately subsequent to the button press even though it was objectively later than that. Stetson et al. (2006) suggested that participant expectations of the relative timing of motor acts and sensory consequences can shift, even to the extent that they can switch places: the later event can be perceived as earlier. This shows that sensory inputs, processed at different speeds but associated with the same event, can be part of one coherent percept.

Multisensory integration in speech perception is the combined use of different sensory modalities in the construction of a speech percept. Most current research on multimodal integration focuses on vision and audition: vision has been demonstrated to enhance the perception of speech when integrated with auditory stimuli in both suboptimal acoustic conditions such as background noise or strong foreign accent (Sumby and Pollack, 1954; Reisberg et al., 1987; MacLeod and Summerfield, 1990) and cases of increased cognitive load such as complicated structure or content (Reisberg et al., 1987; Arnold and Hill, 2001). Visual cues have also been demonstrated to facilitate language acquisition both in children (Mills, 1987) and adults acquiring a second language (Hardison, 2007), and to improve the speech perception of individuals with hearing impairments, especially individuals with cochlear implants (e.g., Geers and Brenner, 1994; Grant and Seitz, 2000; Lachs et al., 2001; Kaiser et al., 2003). Conversely, it has been shown that incongruent visual and auditory cues can modify perception of the acoustic signal in adults (McGurk and MacDonald, 1976; Massaro et al., 1993) and infants (Burnham and Dodd, 1996; Rosenblum et al., 1997). This body of evidence suggests that visual and auditory cues are integrated, along with other cues, in the process of speech perception (Rosenblum et al., 2017).

In recent years, evidence has accumulated demonstrating that tactile information may also be integrated with other modalities in general (e.g., Banati et al., 2000; Lee et al., 2019), and in the perception of speech in particular. In early studies, the effects of tactile information on perception was shown for participants that either had explicit knowledge of the task (Fowler and Dekle, 1991; Gick et al., 2008), or were trained to make a connection between the tactile and the auditory cues (Sparks et al., 1978; Reed et al., 1989; Bernstein et al., 1991). However, later studies have established that tactile information influences auditory perception of uninformed and untrained listeners as well (Gick and Derrick, 2009; Ito et al., 2009; Derrick and Gick, 2013).

Ito et al. (2009) used a robotic device to pull facial skin, creating patterns of facial skin deformation in listeners, that normally accompany the production of the vowels /ε/ and /æ/. They showed that by timing these deformations to auditory stimuli, the perceptual judgments of a synthetic vowel continuum ranging from /ε/to/æ/ were shifted in the expected direction of the bias. For example, when the skin was pulled upward (a deformation consistent with /ε/) the word “head” was preferred, whereas when the skin was pulled downward (consistent with /æ/) the word “had” was preferred. However, deformations applied rearward (orthogonal to directions consistent with vowel production) had no effect on the perceptual judgments. Ito et al. concluded that somatosensory cues can modulate speech perception, but only when these are congruent with those expected in production.

Gick and Derrick (2009) studied the effect of applying air puffs to the back of the hand and the center of the neck at the suprasternal notch on auditory perception of a voicing contrast. In their experiment, native speakers of North-American English were asked to determine whether they heard a syllable with an initial voiceless stop or a syllable with an initial voiced stop. The stimuli, the syllables /ba/, /pa/, /da/, and /ta/ produced by a male native speaker of North-American English, were partially masked by white noise in order to increase ambiguity. During some trials, while the participants heard the stimuli, puffs of air were applied to the back of the participant’s hand, on their suprasternal notch, or as a control beside and tangent to headphones they wore. During the control trials the puff had no direct contact with hair or skin, and was released only into the air near the headphones. The participants were blindfolded; thus, they had no visual information about the application of the air puffs. The duration of the air puffs reflected the duration of the turbulent part of a naturally produced English aspirated consonant. The presence of airflow facilitated the identification of voiceless stops and reduced the identification of voiced stops. Since no such effect was found for the participants in the control group where no direct tactile information was provided, Gick and Derrick concluded that tactile information can modulate speech perception similar to the way vision does.

In a later study, the effect of tactile stimulation of the ankle on auditory perception was tested (Derrick and Gick, 2013). The motivation for using the ankle was two-fold. First, it is a distal location relative to the source of aspiration in typical speaking situations. Thus, while speakers may have experience with feeling air puffs on the back of their hand while they were speaking, or, at least to some extent, with feeling air puffs on the neck while others were speaking, it is unlikely they have similar experience with feeling air puffs on their ankles. Moreover, even if such experience does exist, it is not frequent or robust, thus it is not likely that participants associate the feeling or a puff of air on their ankle with the production of certain speech sounds. Second, the ankle is distant from the ear, and its representation in the somatosensory cortex is distant from the ear’s representation in the somatosensory cortex (Penfield and Rasmussen, 1950). Since comparison of the ankle results to the hand and neck results from Gick and Derrick (2009) did not reveal significant differences, Derrick and Gick concluded that integration is a full-body process and that the association between the felt puff of air and the produced aspirated sound does not depend on direct experience.

Evidence for multimodal speech perception addresses the debate over the nature of the objects of speech perception. From a general auditory point of view (e.g., Klatt, 1979; Stevens, 1981, 1989; Massaro, 1987; Diehl et al., 2004; Hickok and Poeppel, 2007; Yi et al., 2019) the objects of speech perception are sounds. From an ecological or direct perception point of view, represented in the field of speech by Direct Realism (e.g., Fowler, 1981, 1984, 1996), these objects are physical events in the actual world–vocal tract gestures. From the point of view of Motor Theory (Liberman et al., 1967; Liberman and Mattingly, 1985; Liberman and Whalen, 2000) and Articulatory Phonology (Browman and Goldstein, 1986, 1989, 1992; Galantucci et al., 2009) the objects of speech perception are abstract representations of vocal tract gestures rather than physical events as such. The general auditory approaches assume that perception of speech sounds is the same as perception of non-speech sounds. According to this view, the same mechanisms of audition and perceptual learning are used for perception of all types of sounds. Thus, from this perspective, the objects of speech perception may be acoustic or auditory objects, or acoustic landmarks which convey information about the gestures that produced them (Stevens, 2002; Yun et al., 2020). These approaches posit an intermediate representation constructed from sensory input. That is, listeners identify acoustic patterns or features by matching them to stored acoustic representations. In contrast to the non-auditory approaches which assume listeners recover gestures in some form, according to the auditory view listeners perceive “the acoustic consequences of gestures” (Diehl et al., 2004, p. 168) (though see Stevens, 2002). It is assumed that all the relevant information for perception of speech is included in the acoustic signal and is recoverable by general mechanisms of perceptual learning.

But an argument in favor of the non-auditory approaches arises from evidence for multisensory integration, which suggests that the objects of speech perception are distinct from units of non-speech auditory perception [see Goldstein and Fowler (2003) and Rosenblum (2008b) for examples and discussion]. The argument is that if visual or other sensory cues participate in the process of speech perception, the objects of speech perception cannot be auditory, or at least not exclusively auditory, and evidence supporting integration from multiple modalities serves to strengthen this position. However, this argument relies on the interpretation of these experimental findings as supporting multimodal integration in speech perception. For the air puff studies of Gick and Derrick (2009) and Massaro (2009); Derrick and Gick (2013) has argued that it is possible that the participants interpreted the airflow, when it was provided, as aspiration and relied on this interpretation in making their decision. That is, the criticism is that the participants may have based their responses *only* on tactile information without any integration with the auditory cues. The possibility that Gick and Derrick’s findings were simply the result of a general response to tactile stimuli was tested in Gick and Derrick (2009). A tap condition, in which contact with the same test locations was made using a metal solenoid plunger, established that while aero-tactile stimuli were able to shift speech perception, taps on the skin of the participants did not (see supplementary material, Gick and Derrick, 2009). Derrick and Gick (2013) argue that the results of this test are not just a control for a general attention effect caused by the addition of another type of stimuli, but also suggest that the integration of the tactile signal with the auditory signal is dependent upon it being perceived as “event-relevant, as opposed to merely synchronous” (Derrick and Gick, 2013, p. 406).

However, this test does not rule out Massaro’s suggestion that there was no integration, since it is still possible that speech perception during the experiment was unimodal, that is, based solely on aero-tactile information when it was provided, and on auditory information when aero-tactile information was not provided. The stimuli in Gick and Derrick (2009) and Derrick and Gick (2013) were masked by background noise. This made the acoustic stimuli less informative than they could have been under perfect acoustic conditions. Therefore, it might have been the case that the tactile stimulus was the most prominent signal, and as a result a unimodal response was made to it. The current study aims at investigating this question further. Specifically, we use voice onset time (VOT) continua systematically ranging over eight steps from voiceless to voiced sounds rather than endpoint stimuli only (as in the work by Gick and Derrick). This design enables us to show that biasing effects of air puffs are least at the endpoints and greatest for the ambiguous stimuli near the perceptual boundary, supporting interpretation of the tactile cues as forming an integrated rather than unimodal response.

Our prediction is that if integration is not part of the process, then all the sounds along the continuum should be equally affected by aero-tactile cues, and so trials accompanied by air puffs will be perceived unimodally as being voiceless. However, if instead the results show an interaction between the effect of air puff and the effect of step along the continuum this would suggest that aero-tactile information is taken into account along with the auditory information provided, in cases when auditory information is not sufficient for disambiguation, or when the tactile information is not congruent with the auditory information. Such a result would show that participants are using a context-weighted blend of sensory cues in perceiving and categorizing speech sounds, thus providing an example of multi-sensory integration in the perception of speech.

As an additional test for saliency of tactile cues, a continuum consisting of vowel sounds ranging from /ε/ to /ɪ/ in a/hVd/ context was included as a control. While higher vowels are produced with a more constricted oral passage (Jaeger, 1978), both endpoints have approximately equal airflow and are thus not expected to be sensitive to aero-tactile cues. A contrast between an effect of air-puffs on perception of the VOT continua and a lack of it for the vowel continuum would further support an interpretation that cues are integrated only when relevant, that is, that the aero-tactile information is taken into account only in cases where aspiration (or amount of air produced by the speaker) is relevant for the distinction being made.

## Materials and methods

### Participants

In a survey, 42 monolingual native speakers of American English participated in the experiment (24 females; age range 18–56, mean age 28.7, SD = 11.5). Only right-hand dominant participants were recruited. The participants were all residents of Southern Connecticut at the time of the experiment. Their level of education ranged from high school graduates to graduate students. The participants were recruited with flyers and by word of mouth. All were naive to the purpose of the study and had no self-reported speech or hearing defects. All participants provided informed consent overseen by the Yale Human Research Protection Program and were compensated for their time.

### Stimuli

#### Acoustic stimuli

Voice onset time is the interval between the release of a stop consonant and the onset of voicing following or preceding the release (Lisker and Abramson, 1964; Abramson and Whalen, 2017). In American English stops are habitually produced with a positive average VOT. The duration of the positive VOT is longer for voiceless stops than for voiced stops and varies with place of articulation: the more distant the place of articulation from the lips, the longer the VOT. Average VOT durations for American English stops are summarized in Table 1. Note that VOT varies with context: it is shorter for stops when following an obstruent than when following a nasal, a glide, or a vowel. For stops in onset positions it is shortest for those in clusters that begin with /s/ (Randolph, 1989).

**TABLE 1:** Average VOT durations for American English stops (Byrd, 1993).

Place of articulation	VOT length (ms)	
	
	Voiceless	Voiced
Bilabial	44	18
Alveolar	49	24
Velar	52	27

Our endpoint stimuli were taken from a recording of a male monolingual native speaker of American English. He produced six tokens of each of the syllables /pa/,/ba/,/ka/, and /ga/. These were used to obtain his average values for VOT for these utterances. Two eight-step VOT continua were then created, one for the bilabial and one for the velar place of articulation. The continua were created by removing the initial burst from one of the voiceless exemplars (/pa/or/ka/) and then systematically shortening the aspiration in log-scaled steps, with the final step matching the mean aspiration duration of the voiced token. Aspiration durations for each step of the VOT continua appear in Table 2. A non-linear (logarithmic) step size was chosen because psycho-acoustic perception tends to follow Weber’s law (subjective sensation is proportional to the logarithm of the stimulus intensity); e.g., Fastl and Zwicker (2006). See Rosen and Howell (1981) for results on VOT, and Stevens (2000, p. 228) for a similar effect on the perception of duration of burst.

**TABLE 2:** VOT continua steps showing length of retained aspiration at each step (ms).

Step no.	VOT length (ms)	
	
	Bilabial continuum	Velar continuum
1	98	81
2	58	56
3	37	42
4	24	35
5	18	31
6	14	28
7	12	27
8	11	26

An additional continuum consisting of vowel sounds ranging from /ε/ to /ɪ/ in an /hVd/context was included for use as a control. It was synthesized from endpoint recordings of a male monolingual native speaker of North-American English producing “head” and “hid,” by linearly interpolating F1 and F2 values within the vowel over the eight continuum steps, using an iterative Burg algorithm to shift the location of filter poles and zeros in resynthesis (Purcell and Munhall, 2006).^1^

**FIGURE 1:**
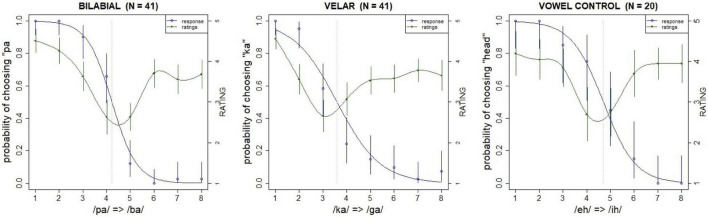
Viability test results for the continua: left scale (blue line) shows probability of choosing voiceless (“pa” or “ka”) or “head” relative to step (dotted vertical line marks 50% crossover point); right scale (green line) shows Likert scale ratings by step. Error bars show 95% confidence intervals.

A pre-test of each continuum conducted online as a Mechanical Turk task was used to assess the quality of the stimuli. The test was run with an independent group of participants that did not take part in the main study (*N* = 41). They were asked to choose whether they heard a “pa” or a “ba” (in the bilabial condition), or “ka” or “ga” (in the velar condition), and rate the goodness of the token on a five step Likert scale. The sounds from the two continua (/pa/-/ba/ and /ka/-/ga/) were presented in the same test. A similar pre-test was conducted for the vowel continuum in which additional 20 participants were asked to choose whether they heard “head” or “hid,” and to rate the goodness of the token. The order of presentation was randomized in both pre-tests. The results of the pretests are plotted in Figure 1.

The bilabial category boundary is approximately centered between its endpoints, that is, its bias (4.2) is close to its midpoint (4.5). The bias was calculated as the 50% crossover point of the psychometric function for the continuum, computed across all listeners. Acuity (a measure of boundary slope) was computed as the difference between the 25 and 75% probabilities for the discrimination function. The velar category boundary is not as centralized and is skewed toward voicelessness (bias = 3.6); that is, longer VOTs were necessary for /ka/ responses. The velar acuity (2.0) is shallower than that of the bilabial (1.1), possibly due to this skew. Finally, the category boundary for the vowel control continuum is also approximately centered (bias = 4.7, acuity = 1.5). The goodness ratings for all three continua are higher at the margins than at the intermediate steps of the continuum, which reflects the fact that the ambiguous sounds were harder to categorize, as expected.

#### Tactile (air puff) stimuli

**FIGURE 2:**
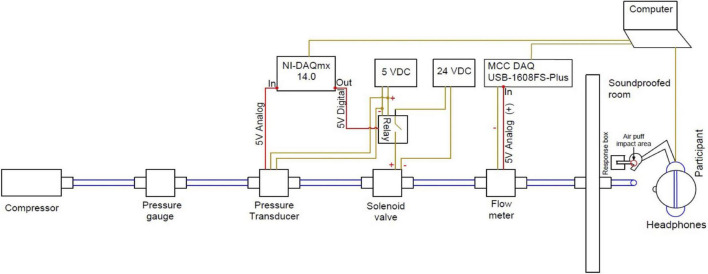
The aero-tactile stimulus presentation system.

To deliver air puff stimuli the following equipment was employed. A three gallon air compressor (Campbell Hausfeld) was connected to a solenoid valve (Parker) used to gate airflow by 1/4-inch polyethylene tubing. The solenoid was toggled by a programmable relay controller device (KMtronic). A pressure transducer (PSC, model 312) and a flow meter (Porter-Parker MPC series) were connected to the tubing in order to monitor pressure and flow data. Solenoid control of airflow, presentation of audio stimuli and data recording were performed using a custom Matlab (Mathworks) procedure that was written for this experiment. The tubing was inserted into a soundproof room through a cable port and stabilized using a table microphone stand (see Figure 2 for a diagram of the system).

**FIGURE 3:**
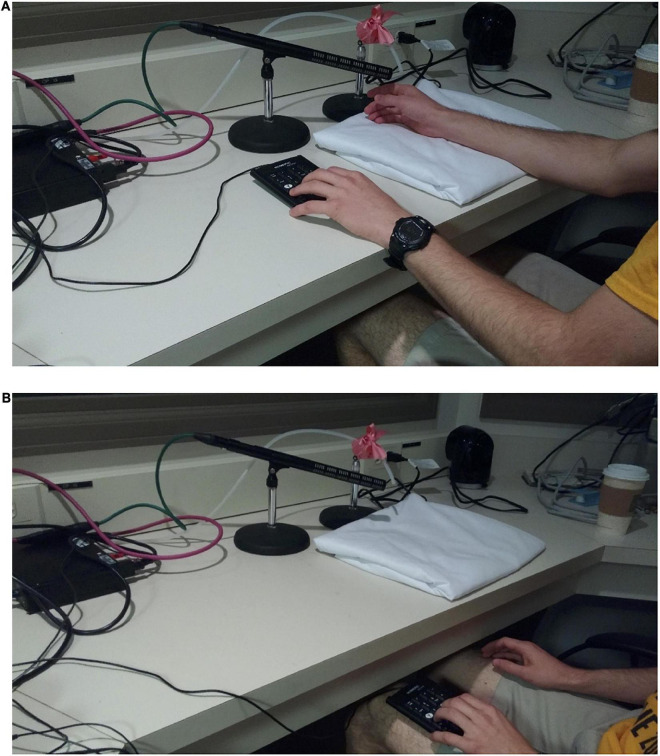
**(A)** Puff delivery setup: participant right hand placed near outflow of airtube, left hand on response button box. Microphone records air puff delivery for verification of timing. **(B)** Puff detection test setup: participant right hand positioned away from outflow of airtube. This test determines whether participant can detect airflow from cues other than tactile hand sensation.

In a given trial the signal to open the air valve solenoid was given by the Matlab procedure, which also controlled acoustic stimulus presentation through the computer’s sound card such that the acoustic onset of each of the stimulus was coincident with the onset of the air puff from the tube. Detectable air turbulence exiting the tube was 87 ms in duration for the bilabial condition and 92 ms in duration for the velar condition. These timings reflect the mean aspiration time (that is, VOT) of the six voiceless tokens that the model speaker produced, thus simulating the temporal properties of the stimuli. The speaker’s mean VOTs fall within the VOT range of initial aspirated stops in American English (54–100 ms, Lisker and Abramson, 1967; Cooper, 1991; Byrd, 1993). The airflow at the exit point of the tube was 5 Standard Liter Per Minute (SLPM). Note that this rate is lower than the average airflow of typical speech (8 SLPM, Isshiki and von Leden, 1964), and significantly lower than the average airflow of voiceless stop consonants in CV syllables (about 56 SLPM, Isshiki and Ringel, 1964). A lower rate was used to better align with the reduction in speed that occurs once aspiration exits the mouth, and additionally to reduce the possibility that the puff would be audible. The exit point of the tube was placed 5 cm away from the participant’s skin, creating an area of initial impact with a diameter of 2–3 cm [similar to Derrick et al. (2009)]. The air puffs were applied on the dorsal surface of the right hand between the thumb and forefinger (see Figure 3A). A microphone placed near the exit of the tube was used to record airflow turbulence during each trial, to verify that air puff stimuli (when scheduled) were delivered with the expected timing.

### Procedure

Each experimental session included two parts, an initial test to verify that the air puffs were felt but not heard, seen or otherwise perceived, and the main part, which tested participant responses to the auditory stimuli in the presence and absence of air puffs. Stimuli were presented to the participants through ear-enclosing headphones (Sennheiser HD 202 II).

#### Puff detection test

In the first part of the experiment the participants heard a short tone (500 Hz, 1,000 ms long) in each trial, which was either followed by a 50 ms long air puff, or not followed by a puff. They were presented with two blocks of 50 trials each, in which 25 of the trials were accompanied by air puffs and 25 were not, presented in randomized order. In the first block the participant’s right hand was located next to the exit of the tube such that they could feel the puff on the back of their hand (see Figure 3A). They were asked to press the “yes” key on a response box with their left hand if they felt or otherwise detected a puff, or the “no” key if they did not. In the second block, the task was the same, but their right hand was positioned on their lap, completely removed from the exit point of the tube (Figure 3B). The goal of this part of the experiment was to verify that the participants felt the puff on their hand but did not hear or see or otherwise detect it. In order to reduce the chances of hearing the puff of air, a small desk fan was used to provide a low level of background noise throughout the experiment. The fan was pointed to the wall and away from the participant. On average this portion of the experiment lasted about 5 min.

#### Perturbed continua testing

In the second part of the experiment, the participant’s right hand was located such that they could feel the puff of air on the back of their hand (Figure 3A). In this part, blocks were presented during which sounds drawn from one of the three continua were tested: from /pa/ to /ba/,/ka/ to /ga/,or /hεd/to/hɪd/. Only one continuum type was used within a given block. Each block included six repetitions of each step of the continuum in randomized order; three instances were accompanied by air puffs and three were not, also randomly ordered. Within a session, each participant heard ten blocks: either five velar blocks and five bilabial blocks, five bilabial blocks and five vowel blocks, or five velar blocks and five vowel blocks, with choices counterbalanced through the participant pool. This resulted in a total of 240 separate judgments [5 blocks × 3 repetitions × 2 puff conditions (−⁣/⁣ +) × 8 continuum steps]. These numbers were chosen on the basis of piloting to keep the session to an approximate 45 min length, and for the same reason participants judged only two of the three possible continua during their session. Overall, 33 were tested for the bilabial continuum, 32 were tested for the velar continuum, and 19 were tested for the vowel control continuum.

Participants were asked to identify the stimulus they heard and to press the corresponding button on a response box on a computer screen: either “P” or “B” to indicate whether they heard /pa/or/ba/ during the bilabial blocks, “K” or “G” to indicate whether they heard /ka/or/ga/ during the velar blocks, and “head” or “hid” to indicate the word they heard during the vowel blocks. They were asked to respond as soon as they had made a decision, but were not constrained in time available for response. The reason for avoiding overt time pressure was our expectation that perceptual decisions involving multimodal stimuli are more difficult, particularly when these are incongruent, as demonstrated by increased reaction times for McGurk studies with mismatched stimuli [see Alsius et al. (2017) for review]. Because we did not have *a priori* knowledge of how puffs could potentially delay formation of an integrated percept and did not wish to truncate that process we opted instead for participant-driven responses.

The presentation order of the continuum auditory stimuli and the accompanying tactile information (puff present vs. absent) were pseudo-randomized throughout each block. The blocks alternated such that there were no consecutive blocks of the same kind. For half the participants, the right button on the response box indicated a syllable with a voiceless consonant (e.g., “pa”). For the other half, the right button indicated a syllable with a voiced consonant. A similar counterbalancing was performed for the vowel blocks. In each trial the Matlab control procedure presented the audio stimulus, gated the air puff (or not), and recorded the participant choices from the response box as well as their response time. New trials began 1 s after each button-press response.

## Results

### Puff detection test

In the first block of the detection test, when their hand was close to the exit point of the tube, participants correctly discriminated puff/no puff conditions at an average rate of 98.1% (s.d. 2.6), with the worst performer at 90%. An exact binomial test confirms that these recognition percentages were well above chance (*p* < 0.01). In the second block, with their hand positioned away from the tube and everything else the same, participants were at chance: 50.4% (s.d. 2.6); best performer 57% (binomial test n.s.). These results confirm that the participants felt the puff of air on their hand, but could not hear, see, or otherwise detect it.

### Perturbed continua testing

**FIGURE 4:**
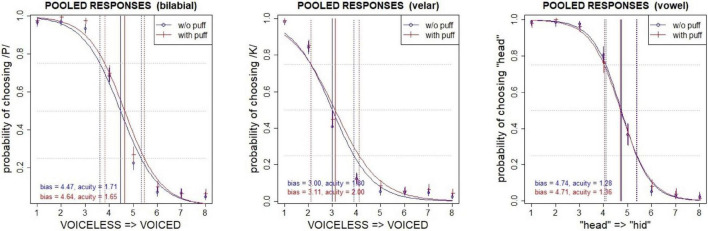
Perceived category boundaries, pooled across speakers, with (red) and without (blue) an air puff. Vertical lines show the bias (50%) crossover, which is systematically shifted in the direction of voiced responses for +puff trials in the bilabial (left) and velar (center) continua, but not in the control vowel continuum (right). 95% confidence intervals are indicated for each pooled response.

In 387 of all trials (1.9%) an air puff was requested but not delivered, or not requested but delivered, due to communication lapses with the solenoid controller. These problematic trials were identified using RMS peaks associated with (or missing from) the puff, measured from an acoustic recording made during the experiment (see microphone in Figure 3A) and were excluded from analysis. Although there was no time pressure to respond, an additional 85 trials were excluded because the button-press response time exceeded 8 s (∼5 s.d.), which was considered sufficiently long that the answer was potentially suspect. The data were then modeled with logistic regression in R (R Core Team, 2018) to estimate the effects of puffs on the perceptual boundary. Figure 4 shows the estimated psychometric functions, pooled across speakers, in the presence and absence of air puffs. The vertical axis represents the probability of choosing a voiceless token or /ε/ (that is, “pa” in the case of the bilabial continuum, “ka” in the case of the velar continuum, or “head” in the case of the vowel continuum). The horizontal axis shows the 8 steps along the continuum. The baseline condition, without puff, is shown in blue lines with circles, and the condition with air puffs is shown in red lines with crosses. Vertical solid lines show the bias (50% crossover point), and vertical dotted lines mark the 25 and 75% probability points along each curve; the distance between these points gives the acuity (a measure of the slope of the boundary). The shift of the bias to the right in the presence of air puffs in the two VOT continua reflects the fact that there were more voiceless responses in this condition; this contrasts with the control vowel continuum which shows no shift in bias under puffs.

#### Quantifying the effect of puffs on perceived categories

A generalized linear mixed-effects model (GLMM) computed with the lme4 package (Bates et al., 2015) was used to assess the significance of the puffs contrast for each of the continua separately as they differ in step size, skewness, and type (the VOT continua were created by manipulating VOT duration, whereas the vowel continuum was created by manipulating formant structure). In this model^2^ the dependent variable (the probability of choosing a voiceless or “head” response) was predicted by the fixed effects of PUFF (−/+) and continuum STEP, with random intercepts by participant ID [random slopes by participant were not supported by model comparison, χ^2^(2) = 0.5094, *p* = 0.775]. The results, summarized in Table 3, show a significant shift under +PUFF for the two VOT continua in the direction of increased judgment of voicelessness (bilabial *z* = 3.16^**^, velar *z* = 2.53*), and no effect of PUFF on the vowel continuum (*z* = −0.31). Marginal *R*^2^ for these models (a measure of effect size), representing the proportion of variance explained by fixed factors alone, was computed using the method of Nakagawa and Schielzeth (2013), as implemented by Lefcheck and Sebastian Casallas (2014). The effect of STEP was significant for all continuum types. The addition of interaction terms for PUFF and STEP did not improve the fit of the model, in all three cases.

**TABLE 3:** Output of the GLMM response model for each continuum.

Continuum	−Air PUFF (baseline) vs. +Air PUFF			
	
	Coefficients	*z*-value	*P*-value	Marginal *R*^2^
Bilabial	0.244	3.160	0.0016**	0.733
Velar	0.216	2.533	0.0113*	0.699
Vowel	−0.037	−0.313	0.7540 n.s.	0.817

For the two VOT continua the effect of +PUFF was to increase the likelihood of a voiceless response; the vowel control continuum was unaffected. R^2^ shows the proportion of variance explained by the fixed factors alone. (**p < 0.01; *p < 0.05.)

#### Comparison of effect sizes for the three continua

In order to compare the relative magnitudes of the puff effect we computed a second GLMM on the data combined from all three continua. In this model^3^ the probability of choosing a voiceless or “head” response was predicted by the fixed effects of PUFF and CONTinuum type and their interaction, and a continuous CSTEP covariate, with random slopes for CONT by participant ID [random slopes for PUFF were not supported by model comparison, χ^2^(3) = 0.4445, *p* = 0.931]. The results are shown in Table 4.

**TABLE 4:** Output of GLMM combining continua to show relative effect sizes (using odds ratios).

	Coefficients	*z*-value	*P*-value	Odds ratios	95% confidence intervals
(Intercept)	7.53485	30.22	0.000	1872.162	(1148.36, 3052.16)
+PUFF	−0.03315	−0.31	0.758	n.s.	
CONTvel	−2.72139	−6.85	0.000	0.066	(0.030, 0.143)
CONTbil	−0.45373	−1.57	0.117	n.s.	
STEP	−1.59468	−66.77	0.000	0.203	(0.194, 0.213)
+PUFF:CONTvel	0.23953	1.76	0.078	1.271	(0.973, 1.659)
+PUFF:CONTbil	0.23953	2.21	0.023	1.360	(1.043, 1.772)

*Marginal R^2^ for this model is 0.756. The interaction terms show the ratio by which the odds ratio of each VOT continuum relative to the Vowel baseline changes for +PUFF, with a larger magnitude observed for the bilabial continuum than the velar.*

For this model the baseline (intercept) encodes the response for −PUFF, Vowel continuum, and CSTEP = 1, and the corresponding odds show the overwhelming preference for voiceless or “head” responses under this condition (1872.2–1). The significant main effect for CONTvel (*z* = −6.85^**^) reflects the leftward skew of the velar continuum (illustrated in Figure 4); i.e., in the direction of increased voiced responses over baseline. The continuous CSTEP covariate (continuum step) has the expected negative correlation with stimulus VOT and vowel quality (voiceless > voiced, “head” > “hid”). Because of the inclusion of the non-responsive vowel control, the overall effect of +PUFF is not significant, but its interactions with the two VOT continua show significant positive shifts in the direction of increased voiceless responses over baseline (velar *z* = 1.76, bilabial *z* = 2.27*). This is confirmed through *post hoc* (Tukey HSD) comparisons of +PUFF > −PUFF, which show velar *z* = 2.48* and bilabial *z* = 3.35^**^. The odds ratios for these interactions show the ratio by which the odds ratios for the main effects (CONTvel/CONTvow, CONTbil/CONTvow) changes for +PUFF; i.e., their relative increase over baseline. Interpreted as an effect size this indicates that +PUFF had a greater effect on the bilabial continuum (odds ratios = 1.36) than the velar continuum (odds ratios = 1.27); however, the 95% confidence intervals overlap for these values, and the significance within this model for the velar interaction is marginal.

#### Analysis of individual results

To assess the degree to which individual participants were sensitive to the air puff effect we computed separate logistic regression models for each, with response predicted by the fixed effect of PUFF and STEP as a continuous covariate.^4^ About two thirds of the participants who heard the bilabial continuum showed a shift toward increased probability of voiceless responses (23/33; binomial test *p* < 0.02), as did about three quarters of the participants who heard the velar continuum (24/32; binomial test *p* < 0.01). About half of the participants who heard the vowel continuum showed small and non-significant shifts toward ‘head’ responses (9/19; n.s.). See Table 5 for summary statistics.

**TABLE 5:** Summary of the individual models computed for the participants.

Continuum	Mean coefficient	s.d. of coefficient	Range of coefficient
Bilabial	0.26766	0.479	−0.87388: 1.66863
Velar	0.21979	0.546	−0.83977: 0.98542
Vowel	−0.00845	−0.548	−0.99308: 1.02929

#### Analysis of response times

**FIGURE 5:**
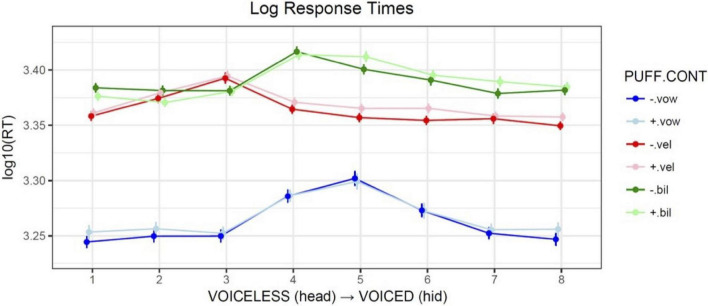
Comparison of mean log_10_ response times averaged across participants, by PUFF (+airflow vs. –airflow), CONTinuum type (VOWel, VELar, BILabial), and continuum STEP. Error bars show the standard error of the mean.

Response times were measured as the duration in milliseconds from the onset of the audio stimulus (which was coincident with the start of the air puff, if present), to the button-press event. For analysis they were log-scaled in order to normalize a right-skewed distribution. Figure 5 illustrates the mean response times pooled across participants, by PUFF, CONTinuum type, and STEP along the continuum. An overall effect of CONTinuum type was observed, with bilabial responses slower than velar responses in general, and both significantly slower than vowel control responses.

A linear mixed-effects model^5^ computed using lme4 with significance assessed using the lmerTest package (Kuznetsova et al., 2017) in R was used to predict the log_10_ response time from the fixed effects of PUFF, CONTinuum, and (discrete) continuum STEPs. Model comparison supported the complete interaction between fixed factors and the inclusion of random slopes and intercepts for each by participant. The analysis modeled discrete rather than continuous steps along the continuum to investigate how response time interacted with stimulus, with the expectation that responses to stimuli in the ambiguous range of each continuum would be slower. Significant results are shown in Table 6.

**TABLE 6:** Output of LMM predicting log_10_ response times from PUFF, CONTinuum, and stimulus STEP along the continuum.

	Coefficients	*t*-value	*P*-value	Significance
STEP4	0.04363	6.248	0.000	***
STEP5	0.06053	8.575	0.000	***
STEP6	0.03054	4.246	0.000	***
CONTvel	0.1026	14.307	0.000	***
CONTbil	0.1275	18.295	0.000	***
+PUFF:CONTbil	−0.01717	−2.163	0.031	*
STEP3:CONTvel	0.02652	3.248	0.001	**
STEP4:CONTvel	−0.03783	−4.552	0.000	***
STEP5:CONTvel	−0.06216	−7.539	0.000	***
STEP6:CONTvel	−0.03499	−4.247	0.000	***
STEP8:CONTvel	−0.01404	−1.729	0.084	
STEP5:CONTbil	−0.04550	−5.550	0.000	***
STEP6:CONTbil	−0.02480	−3.022	0.003	**
STEP7:CONTbil	−0.01666	−2.049	0.040	*
+PUFF:STEP5:CONTbil	0.03088	2.759	0.006	**
+PUFF:STEP6:CONTbil	0.0214	1.911	0.056	
+PUFF:STEP7:CONTbil	0.02473	2.210	0.027	*

The baseline represents −PUFF at STEP1 on the Vowel continuum. Only significant values are shown. Pseudo-R^2^ for this model (comparison of fitted vs. observed values) is 0.447. (***p < 0.001; **p < 0.01; *p < 0.05).

The pattern of main effects confirms that response times are slower for the ambiguous intermediate steps (4, 5, 6), and that responses for the two VOT continua are slower overall than for the vowel control baseline, with the bilabial responses slower than the velar. The interaction of STEP with the velar continuum reflects its left-skewed crossover, such that step 3 (closest to the boundary and thus most ambiguous) is significantly slower, while subsequent steps are faster relative to baseline. The negative coefficient for the interaction of +PUFF and the bilabial continuum suggests an overall facilitation effect (responses are faster than baseline), which Figure 5 suggests is active on the voiceless end of the continuum (steps 1, 2). This effect was likely due to the congruent nature of the added information, namely, consistent with what would be felt on the hand if placed near the mouth during production of a voiceless stop. The interaction of steps 5, 6, and 7 with the bilabial continuum shows that these responses were significantly faster than baseline *without* puffs, and significantly slower than baseline *with* puffs, indicating that over this portion of the continuum puffs represented an incongruent and thus inhibitory distraction, perhaps because of the mismatch of consistent puff duration with reduced aspiration for these tokens. Similar reaction time effects of secondary information for unambiguous portions of a continuum have been previously reported (e.g., Whalen, 1984). The differential effects of air puffs on response times argue against a unimodal effect and instead suggest that tactile cues are weighted according to both relevance and congruence.

## Discussion

The current study found that presence of air puffs significantly increased the likelihood of choosing voiceless responses for the two VOT continua, and consequently the category boundaries for both VOT continua were shifted toward the voiced end of each continuum in the presence of air puffs. The effect was found to be larger for the bilabial continuum than for the velar continuum, though not significantly so. The observed difference may be due to the unbalanced (left-skewed) velar continuum. Air puffs had no effect on choices for the control vowel continuum.

In this work VOT continua were used rather than endpoints alone to address the critique raised by Massaro (2009). Gick and Derrick (2009) and Derrick and Gick (2013) used CV exemplars presented in background noise. Because this degraded acoustic signal might not be sufficient for categorization listeners could be simply disregarding it, and instead be relying solely on the presence or absence of the aero-tactile cue. However, the current study shows that an air-puff alone in each trial was not sufficient for deciding the category, and that listeners instead weighted the tactile cue both by relevance (no effect on the vowel continuum) and quality of the auditory signal (minimal effects at endpoints, maximal effects near the ambiguous crossover point of the VOT continua). While the presence of an air puff did result in more voiceless responses, these acted to shift the existing perceptual boundary rather than overriding it; in other words they did not uniformly increase voiceless responses at every continuum step. This suggests that aero-tactile sensation was processed as a potential additional cue for disambiguation of voiceless from voiced sounds, but weighted by relevance and the degree of ambiguity, as an instance of true multi-sensory integration.

Although participants were not instructed to answer as quickly as possible, analysis of response times did reveal significant differences between continua and within continua. The intermediate steps of the continua, that is, the ambiguous stimuli between the two endpoints, were the hardest for participants to categorize, as expected. This was suggested by the longer response times associated with these steps, for all three continua, as longer response times generally indicate a greater cognitive load (e.g., DeLeeuw and Mayer, 2008). For the two VOT continua in general response times were slower than the vowel control baseline. It is important to note that the response times for the VOT continua did not show a uniform response to air puffs, shown most clearly by the bilabial continuum. As illustrated in Figure 5 and shown by the results in Table 6, air puffs had a *facilitatory* effect at the voiceless end of the continuum (encoded by the negative + PUFF:CONTbil interaction; *t* = −2.2*); i.e., responses were faster with puffs. This effect was likely caused by the complementary nature of the added information (cf. Whalen, 1984). Conversely, air puffs at the voiced end of the continuum had an *inhibitory* effect [encoded by the positive + PUFF:STEP:CONTbil interaction for steps 5 (*t* = 2.8^**^), 6 (*t* = 1.9⋅), and 7 (*t* = 2.2)]. In this case, the added information was incongruent. As no overall main effect of PUFF was observed, these results are inconsistent with Massaro’s position that listeners respond to air puff stimuli unimodally; rather, the pattern of results indicates that an air puff cue is evaluated together with the concurrent audio stimulus and weighted by the ambiguity of the latter.

We have mentioned, in the Introduction, that evidence for multisensory integration has been used to argue in favor of certain approaches for the objects of speech perception. The argument was that if non-acoustic information, tactile in the current case, is an integral part of the process of speech perception, the objects of speech perception cannot be auditory, or at least not exclusively auditory. A counter argument that has been discussed in the literature is that the association with visual and other sensory information may be learned, that is, associated by experience with the auditory primitives (e.g., Massaro, 1987; Diehl and Kluender, 1989; Kluender, 1994). Rosenblum (2008a) offers a few arguments against learned association: first, multisensory integration has been shown in pre-linguistic infants (Rosenblum et al., 1997). Second, multisensory integration has been shown to operate at an early stage of online perception, before phonetic categorization and possibly before phonetic feature extraction (Summerfield, 1987; Green, 1998; Rosenblum and Gordon, 2001; Choi et al., 2021). The evidence for multisensory integration at an early stage of speech processing is consistent with evidence for multisensory integration in other domains [for discussion see Shimojo and Shams (2001); Stoffregen and Bardy (2001); but see Remez et al. (1998)]. Multisensory integration has been shown in contexts where participants had no speech experience associated with the task (Fowler and Dekle, 1991). However, in the experiment conducted by Fowler and Dekle the participants were aware of the task and thus it is not clear that this is indeed a counter-argument for learned association.

Based on the evidence cited above, Rosenblum (2008a) argues that the objects of speech perception are modality-neutral. Specifically, he argues for gestural objects that have spatial and temporal dimensions but are not specified along any sensory dimension. According to this view the sensory dimensions are the medium through which perceivers recover the gestures, and the objects of speech perception themselves are of a higher order than just auditory, visual or tactile. The idea is that perception is sensitive to underlying gestural primitives instantiated in any modality. This view, which is consistent with Direct Realism, Motor Theory and Articulatory Phonology, is supported by the cited evidence for the automaticity and ubiquity of multisensory integration. However, it is not the only view that is consistent with such evidence. It may be the case that the objects of speech perception do have a sensory content, but they are specified for more than one modality. That is, it may be the case that they are not just auditory, but multimodal in nature. The evidence presented here suggests that tactile information is considered during the perception of speech [and see as well Bruderer et al. (2015) and Choi et al. (2021)]. However, it does not rule out the possibility that the integration of the additional tactile modality operates in later stages of online perception.

The lack of an obvious connection between distal aero-tactile stimulation and speech perception in the current experiment contrasts with the direct somatosensory link posited by Ito et al. (2009). In their experiment they determined that perception of vowels is affected by deforming the skin on the face of the participant in the same way the skin moves when these vowels are produced. Crucially, deformations applied orthogonal to the up and down directions used in the production of these vowels had no effect. This kind of direct link between somatosensory stimulus and speech perception is not reflected in the current study, as air puffs were applied on the back of hand of the participants, a location that does not typically relate directly to the tactile sensation of aspiration during the production of stop consonants. Nonetheless, the results presented here confirm that aero-tactile stimulation can also shift perception, though only when the cue is relevant (vowel perception was unaffected) and the primary VOT cues are ambiguous. In both the air puff and skin pull studies then, tactile information affected speech perception only when the cues applied were congruent with the ones expected in production of the perceived sounds.

In addition to addressing Massaro’s critique against Gick and Derrick (2009) and Derrick and Gick (2013), and providing evidence for integration of auditory and tactile input in the perception of speech, the current work extends the work of Gick and Derrick in two additional ways. First, rather than a between-subject design, here a within-subject design was used in which each participant served as their own control. Thus, the comparison between the perception of the VOT continua with and without tactile stimuli was done within participant, and not across groups of participants. This allowed a direct comparison between the responses of the same individual to the same auditory stimuli with and without aero-tactile stimulus. Second, a vowel continuum was used as a control. Since aero-tactile sensation is hypothesized not to be relevant for distinguishing /ε/ from /ɪ/, effects observed on the VOT continua but not on the vowel continuum shows that the obtained results were not just an artifact of puffs alone, but rather a context-sensitive effect, indicating a true multi-sensory phenomenon. Moreover, since this was a within-subject design, the comparison between the VOT continuum and the vowel continuum was done within participant. That is, the participants that heard vowel blocks were sensitive to the effect of aero-tactile stimulation when the acoustic stimuli were taken from a VOT continuum, and at the same time showed no such sensitivity when the acoustic stimuli were taken from a vowel continuum. As discussed above, these results are consistent with Ito et al. (2009) showing that while tactile cues can indeed modulate perception, they do so only when congruent with the production contrast being disambiguated.

While statistically significant, the effect of puffs found in this study was not observed for all the participants, similar to other studies of multimodal integration. Population estimates of audiovisual integration susceptibility vary widely and range between 26 and 98% of the tested population (Nath and Beauchamp, 2012). In the current study, between two thirds (in the bilabial continuum) and three quarters (in the velar continuum) of the participants showed susceptibility to puffs in their responses. These clear majorities contrast with participants who showed some effect of puff on their response to the vowel continuum (about half), though of these shifts, none were significant. The absence of effect on the VOT continua for some of the participants may stem from lack of sufficient statistical power, given the small size of the effect and further division of the data into participant-sized bins, though for most of the participants a significant effect was found even after the division of the data. Finally, it is possible that some of the participants were not affected by the aero-tactile stimuli because of the relatively low airflow (5 SLPM), in comparison to the average airflow of voiceless stop consonants in CV syllables (about 56 SLPM, Isshiki and Ringel, 1964). Although the puff detection test has confirmed that these participants have felt the puff, it is possible that they did not interpret it as related to aspiration since the airflow was inconsistent with the typical airflow of speech.

The current study did not test the length of the integration window, as it did not vary the relative timing of the auditory stimuli and the tactile stimuli. However, it has been shown previously that this window operates asymmetrically. Derrick et al. (2009) and Gick et al. (2010) found for audio-tactile stimuli that integration extends to 200 ms when air puff follows audio but only 50 ms when air puff precedes audio. Bicevskis (2015) studied visuo-tactile integration by presenting participants with video of faces producing the syllables /pa/ and /ba/, without an air puff, or accompanied by an air puff occurring synchronously with the visual stimuli or at different timings, up to 300 ms before and after the stop release. Bicevskis found that the integration window for visuo-tactile stimuli is also asymmetric: when an air puff followed visual stimuli the integration window extended to 300 ms, but when it preceded visual stimuli the integration window only extended to 100 ms. These windows extend farther than the audiovisual integration window reported by Munhall et al. (1996) for McGurk phenomena (0–180 ms) and also Van Wassenhove et al. (2007) (−30 to 170 ms) but exhibit the same properties of asymmetry. The asymmetry appears to be ordered by the relative speed by which each modality is processed: visual input is processed more slowly than auditory (Molholm et al., 2002), and tactile sensation is also slower than audition. Munhall et al. (1996) suggest that knowledge of the natural world may play a role in validating the range over which integration is permitted to occur; e.g., thunder is expected to follow lightning, and air turbulence is typically heard before it is felt. Thus, relative timings of potential speech cues that violate these expectations are potentially less likely to be integrated.

The tolerance for asynchrony in multimodal integration differs from that observed for parsing the acoustic signal alone. For example, work by Remez and colleagues confirms that individual tones in sinewave speech are not separate streams needing integration but are instead necessarily tightly timed (within 50 ms) in order to provide speech information Remez et al. (2008, 2010). Similarly, if a non-speech “chirp” in a duplex paradigm precedes a speech third formant (F3) by more than 50 ms, the non-speech percept generally “captures” the F3, leaving the ambiguous base as the percept (Whalen and Liberman, 1996). For multimodal integration, the tolerances are greater, presumably due to the need to buffer separately acquired channels with differing inherent timescales for perception.

The limited activation of speech percepts by the puffs themselves further argues for an integrative rather than a unimodal biasing process. In audiovisual integration, it is clear that both channels can convey the speech signal at greater than chance levels independently, though not to the same degree (phonemes > visemes), and their respective weighting in combination can vary with ambient factors such as background noise (Vatikiotis-Bateson et al., 1998). Because the tactile sensation of puffs alone has insufficient bandwidth to convey anything like the full speech signal, its potential effects are limited to those restricted cases where acoustic ambiguity lowers the threshold for such cues to become relevant in producing an integrated percept.

The mismatch in bandwidth capacity, processing speeds and tolerance for asynchrony suggests that some form of perceptual buffering exists for each contributing modality, which is then weighted to form the composite percept (Rosenblum et al., 2017). But although we have observed an effect of distal aero-tactile stimulation on speech perception, we have not provided an explanation for why the phenomenon occurs. Numerous studies have shown that listeners can make use of all available information, “parsing” it into plausible percepts (Fowler and Smith, 1986; Fowler and Brown, 2000) and rejecting components that do not parse as being simultaneous non-speech (Xu et al., 1997). Multimodal integration indicates that such speech parsing goes beyond the acoustic signal to include all aspects of the production (Liberman and Mattingly, 1985; cf. MacDonald, 2018). There is considerable evidence that much of this integration can occur before much, if any, experience has been attained [supported by Bruderer et al. (2015)]. Still, if familiarity plays a role in the uptake of multisensory information, the use of tactile (puff) information is puzzling. It is possible that close proximity of the hand to the mouth during the babbling phase of language acquisition might develop a learned association between aspiration and tactile sensation felt there. Similarly, such association may also arise from exposure as children to speech produced by others who are in close proximity to them. Hall (1966) defined four spaces encircling every person. The most inner space, the intimate space, is characterized as the spaces closest to the body, up to 45 cm away from it. This is a space reserved for sexual partners and children. This distance is sufficiently short for aspirated stops to be felt on the skin of a child or a partner. Children are also found in close proximity to others during social interaction with their peers: Aiello and Jones (1971) studied the proxemic behavior of children ages 6–8 and found that the mean distance between children during social interaction differed by sex and sub-culture, but overall ranged between 5.3 and 13.5 inches, a distance sufficiently short for aspirated stops to be felt on the skin. Aiello and De Carlo Aiello (1974) found that personal space grows bigger as children grow older, suggesting that the chance of being exposed to felt aspiration at younger age is larger than it is in conversations at later stages of life. Because such stimulation would not be particularly localized to a single point of contact, the association between aspiration and tactile sensation could then eventually be generalized to any skin location, consistent with Gick and Derrick (2009) and Derrick and Gick (2013) who show that air puffs affect VOT perception when the point of contact is the neck or even the ankle. They also show that not just any tactile stimulus produces the effect, as tapping the skin at the same location as delivered air puffs did not affect perception, and this selective response suggests some type of learned link between aspiration and air puffs rather than a general tactile effect. However, while the pathway to acquiring an association between VOT aspiration and the tactile sensation specific to feeling its effect on the skin is speculative, the results from Gick and Derrick (2009) and this confirmatory study indicate that such an association is real. Once available, tactile information joins other potential cues (visual, lexical, etc.) available for exploitation by language users to disambiguate the speech signal.

## Data Availability Statement

The raw data supporting the conclusions of this article will be made available by the authors, without undue reservation.

## Ethics statement

The studies involving human participants were reviewed and approved by Yale Human Research Protection Program. The participants provided their written informed consent to participate in this study.

## Author contributions

DG constructed the experimental setup and collected the data. MT wrote the controlling software and performed data analysis. All authors designed the research, wrote the manuscript, and approved the submitted version.

## Conflict of Interest

The authors declare that the research was conducted in the absence of any commercial or financial relationships that could be construed as a potential conflict of interest.

## Publisher’s Note

All claims expressed in this article are solely those of the authors and do not necessarily represent those of their affiliated organizations, or those of the publisher, the editors and the reviewers. Any product that may be evaluated in this article, or claim that may be made by its manufacturer, is not guaranteed or endorsed by the publisher.

## Footnotes

1 The 24 sound files used as acoustic stimuli are available as Supplementary Material from https://tinyurl.com/2p8tjfnh.

2 glmer (RESP∼PUFF + STEP + (1| ID), family = binomial).

3 glmer[RESP∼PUFF * CONT + CSTEP + (1 + CONT| ID), family = binomial].

4 glm (RESP∼PUFF + CSTEP, family = binomial).

5 lmer [LRT∼PUFF * STEP * CONT + (1 + PUFF + STEP + CONT| | ID)].
